# SPIN: sex-specific and pathway-based interpretable neural network for sexual dimorphism analysis

**DOI:** 10.1093/bib/bbae239

**Published:** 2024-05-28

**Authors:** Euiseong Ko, Youngsoon Kim, Farhad Shokoohi, Tesfaye B Mersha, Mingon Kang

**Affiliations:** Department of Computer Science, University of Nevada, Las Vegas, Las Vegas, NV, USA; Department of Information and Statistics and Department of Bio&Medical Bigdata (BK21 Four program), Gyeongsang National University, Jinju, Republic of Korea; Department of Mathematical Sciences, University of Nevada, Las Vegas, Las Vegas, NV, USA; Department of Pediatrics, Cincinnati Children’s Hospital Medical Center, University of Cincinnati, Cincinnati, OH, USA; Department of Computer Science, University of Nevada, Las Vegas, Las Vegas, NV, USA

**Keywords:** Interpretable deep learning, SPIN, Sexual dimorphism analysis, Cancer, Asthma

## Abstract

Sexual dimorphism in prevalence, severity and genetic susceptibility exists for most common diseases. However, most genetic and clinical outcome studies are designed in sex-combined framework considering sex as a covariate. Few sex-specific studies have analyzed males and females separately, which failed to identify gene-by-sex interaction. Here, we propose a novel unified biologically interpretable deep learning-based framework (named SPIN) for sexual dimorphism analysis. We demonstrate that SPIN significantly improved the C-index up to 23.6% in TCGA cancer datasets, and it was further validated using asthma datasets. In addition, SPIN identifies sex-specific and -shared risk loci that are often missed in previous sex-combined/-separate analysis. We also show that SPIN is interpretable for explaining how biological pathways contribute to sexual dimorphism and improve risk prediction in an individual level, which can result in the development of precision medicine tailored to a specific individual’s characteristics.

## INTRODUCTION

The prevalence, course and severity of several complex diseases, including cancer, asthma, coronavirus and autoimmune disease, differ by sex [1–4]. For instance, cancer incidence involving colorectal, stomach and liver is higher in males than in females; bladder cancer and leukemia have been predominantly more common in males than in females [3, 4]. Boys are also twice as likely to develop asthma as compared with girls [5]. Although differences in lifestyle and hormones have been put forward as explanations for the sex bias in these diseases, the role of genetic factors in sexual dimorphism is historically understudied. Most genetics and clinical outcome studies have been mainly analyzed in sex-combined frameworks in which sex is often considered as a covariate for secondary data analyses [6–8]. Although sex-combined analysis frameworks may increase sample size and power for identifying risk factors with similar effect directions between the sexes, such practices may reduce the power to detect the effects of opposite direction between males and females due to a net canceling effect. Consequently, there are gaps in our understanding of the biological differences and mechanisms that underlie sex-associated disease prevalence and treatment.

Few studies have addressed sexual dimorphism using naïve approaches that analyze genomic data (e.g. genome-wide association studies, gene expression) of male and female groups separately [7, 9, 10]. However, these studies performed the sex-specific analysis on males and females separately, which assumes that males and females have independent biological mechanisms. Consequently, it often does not identify the gene-by-sex interaction (GxSex) that represents relationships among genes between sexes. GxSex studies are critically needed to elucidate patient stratification based on their individual genotypes and expression signatures and to understand implications of sexual dimorphism. Without conducting GxSex analysis, the current sex-specific analysis frameworks fail to identify high-risk individuals or vulnerable groups.

A wide range of potential approaches could help to address the methodological challenges for sexual dimorphism analysis, including a unified interpretable deep learning (DL) framework. A unified DL framework has the following major advantages for sexual dimorphism analysis. First, it accommodates a learning process in which male and female samples are simultaneously learned by a single joint model, where both sex-specific and -shared biological mechanisms can be identified. Secondly, the unified learning approach increases the sample size along with the aggregation of male and female samples, which leads to improving predictive power with robust interpretable DL models. Lastly, the unified DL framework captures complex nonlinear relationships among features. DL models learn multi-level representations by composing multiple layers of functions that automatically recognize optimal feature representations to capture nonlinear relations between biological entities (i.e. genes/pathways) and clinical outcomes.

Interpretable DL models can identify significant biological factors for sexual dimorphism analysis, opposite to conventional DL models of the black-box nature, which make the predictive mechanism difficult to interpret. As an intrinsic interpretable DL approach, a pathway-based DL model embeds relationships between genes and pathways in a model architecture, which enhance the DL interpretability and model robustness [11–15]. The interpretability of DL models could be further performed by two approaches: global and local interpretations. Global interpretation examines *what* significant factors are involved in a biological/clinical disease at a population level, whereas local interpretation analyzes *how* the significant factors affect prediction at an individual level. To the best of our knowledge, there has not yet been a unified interpretable DL framework for sexual dimorphism analysis.

**Figure 1:**
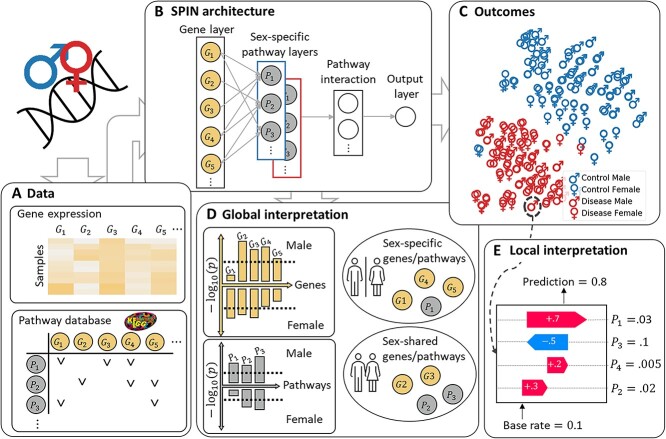
Overview of our proposed method, SPIN’s neural network architecture and analyses. (A) The data to train SPIN for sexual dimorphism analysis: gene expression and pathway database. (B) The graphical representation of our unified interpretable neural network based framework. SPIN has sex-specific pathway layers: male-specific (in the blue box) and female-specific layers (red box). (C) The visualization of the SPIN’s outcomes. SPIN shows discrepancy of clinical outcomes between the sexes (male and female symbols) as well as between diseases (in red) and control (in blue) samples. (D) SPIN identifies statistically significant sex-specific and -shared genes/pathways at a population level. (E) SPIN is interpretable to understand biological mechanisms of how the pathways have positive/negative effects on a prediction at an individual level.

Here, we developed a novel unified biologically interpretable DL framework, called Sex-specific and Pathway-based Interpretable Neural Network (SPIN), for sexual dimorphism analysis (Figure 1). SPIN incorporates a biological knowledge of the relationships between genes and pathways in its architecture for the capability of intrinsic biological interpretability and predicts clinical outcomes, such as stages of a cancer, prognosis prediction and survival analysis. SPIN not only achieves higher prediction performance than the existing sex-combined and -specific analysis models, but also identifies sex-specific and -shared biomarkers leading to potential findings for prognosis and treatment in complex diseases. Specifically, the SPIN’s global interpretation identifies significant sex-specific and -shared genes/pathways based on the distributions of entire samples. On the other hand, the local interpretation analyzes processes of how the model produces predictions on an individual sample or subgroups of interest, rather than understanding general mechanisms of the whole population. The SPIN framework offers the following contributions: (1) it remarkably improves its predictive performance compared with other benchmark models, (2) it identifies sex-specific and -shared biological risk factors nonlinearly associated with the clinical outcomes and (3) it provides insight into further understanding of biological processes for each individual. This study considers two case studies, focusing on survival analysis (cancer) and risk score prediction (asthma).

## MATERIAL AND METHODS

### SPIN design

SPIN is a hierarchically multi-layered network consisting of (1) the gene layer, (2) the sex-specific pathway layers, (3) the hidden layers and (4) the output layer. The architecture of SPIN represents hierarchical biological entities and their interactions. In SPIN, gene expression profiles (e.g. RNA-Seq) on male and female samples are fed into the gene layer of the model, followed by sex-specific pathway layers that represent a set of biological pathway entities as high-level representations of pathway’s activation. The two pathway layers represent sex-specific processes of pathways corresponding to male and female groups, which are capable of identifying significant sex-specific and -shared risk factors. The sex-specific pathway layers are sparsely connected to the hidden layer in which each node encodes biological interactions between pathways. The sparse connections are shared for both male- and female-specific pathway layers toward the hidden layer, constrained by the sparse coding. The output layer predicts target outcomes associated with biological problems. SPIN is a general framework for sexual dimorphism analysis due to its flexible model design applicable to diverse biological problems.

#### The gene layer

The gene layer introduces gene expression data as the input to SPIN. Let gene expression data be denoted as $\boldsymbol{G} \in \mathbb{R}^{n \times q}$, where $n$ and $q$ are the numbers of samples and genes, respectively. The nodes in the gene layer represent values for a set of genes $\boldsymbol{G}=\{\boldsymbol{g}_{i}: \forall _{i} \in 1, \ldots ,q\}$, $\boldsymbol{g}_{i} \sim \mathcal{N}(0,1)$.

#### The sex-specific pathway layers

The sex-specific pathway layers represent the activations of male- and female-specific biological pathways (i.e. pathway enrichment). The set of genes in the gene layer connects to the sex-specific pathway layers for pathway-based interpretation and sexual dimorphism analysis. The connections are constrained to be sparse by the gene set annotation, such as KEGG. To implement the sparse connections between the gene and the sex-specific pathway layers, we generate a mask matrix that reflects the gene–pathway relationships. The mask matrix is a binary bi-adjacency matrix, $\boldsymbol{M}_{G}=\{m_{ij}|1 \leq i \leq q$, $1 \leq j \leq r\}$, where an element $m_{ij}$ is one if $i$-th gene belongs to $j$-th pathway; otherwise it is zero. $r$ is the number of pathways. To differentiate characteristics of pathway enrichment on males and females, SPIN incorporates the sex-specific weights matrices, $\boldsymbol{W}_{G}^{M} \in \mathbb{R}^{q \times r}$ for men and $\boldsymbol{W}_{G}^{F} \in \mathbb{R}^{q \times r}$ for women, while the male-/female-specific pathway layers share the same list of pathways. The sex-specific pathway layers $\boldsymbol{P} \in \mathbb{R}^{n \times r}$ are computed as 


(1) \begin{align*}& \boldsymbol{P}= \left\{ \begin{array}{@{}llc} \boldsymbol{P}^{M}=\phi(\boldsymbol{G}(\boldsymbol{W}_{G}^{M} \odot \boldsymbol{M}_{G}) + \boldsymbol{b}_{G}^{M}) & \text{if male},\\ \boldsymbol{P}^{F}=\phi(\boldsymbol{G}(\boldsymbol{W}_{G}^{F} \odot \boldsymbol{M}_{G}) + \boldsymbol{b}_{G}^{F}) & \text{otherwise}, \end{array}\right.\end{align*}

where $\boldsymbol{b}_{G}^{M}, \boldsymbol{b}_{G}^{F} \in \mathbb{R}^{n \times 1}$ are sex-specific bias vectors, $\phi (\cdot )$ is a nonlinear activation function (i.e. ReLU in this study), and $\odot $ stands for an element-wise multiplication operator.

#### The hidden layers

The hidden layers represent the interaction effects of a set of pathways, shared by sex. The sex-specific pathway layers infer pathway enrichment that may differ by sex, whereas the hidden layers capture common hierarchical biological mechanisms of multiple pathways regardless of sex. The nodes in the hidden layers, $\boldsymbol{H} \in \mathbb{R}^{n \times s}$ ($s$ is the number of hidden nodes), are computed by 


(2) \begin{align*}& \boldsymbol{H}= \left\{ \begin{array}{@{}ll@{}} \boldsymbol{H}_{1}=\phi(\boldsymbol{P}(\boldsymbol{W}_{P} \odot \boldsymbol{M}_{P}) + \boldsymbol{b}_{P}) & \text{if}\ l=1\\ \boldsymbol{H}_{l}=\phi(\boldsymbol{H}_{l-1}(\boldsymbol{W}_{l-1} \odot \boldsymbol{M}_{l-1}) + \boldsymbol{b}_{l-1}) & \text{otherwise} \end{array}\right.,\end{align*}

where $\boldsymbol{H}_{1}$ is the first hidden layer, $\boldsymbol{H}_{l}$ is the $l$-th hidden layer ($l \geq 2$), $\boldsymbol{W}_{P} \in \mathbb{R}^{r \times s}$, $\boldsymbol{M}_{P} \in \{0, 1\}^{n \times 1}$ and $\boldsymbol{b}_{P} \in \mathbb{R}^{n \times 1}$ are a weight matrix, a mask matrix and a bias vector, respectively. The mask matrices are optimized by sparse coding to infer hierarchical interactions among pathways, whereas the sparse connections between the gene layer and the pathway layer are given by pathway databases. The detail of the sparse coding algorithm is in the supplementary document (S1. Sparse coding).

#### The output layer

The output layer produces predictions of target problems. SPIN can be optimized for various biological problems, as a general framework of sexual dimorphism analysis. For instance, the output layer consists of a node for regression or binary classification problems (e.g. survival analysis, binary classification for risk score prediction), while it includes multiple nodes corresponding to the class labels with the softmax activation in the output layer (e.g. cancer stage classification or disease phenotype classification). In this study, we included a node in the output layer for both survival analysis and risk score prediction. For the survival analysis, SPIN generates a Prognostic Index (PI), which is introduced to the Cox Proportional Hazards regression model (Cox-PH). The outcome of the survival analysis is obtained without a bias node according to the Cox-PH model’s design by 


(3) \begin{align*}& \boldsymbol{Z}=\boldsymbol{W}_{H} \boldsymbol{H},\end{align*}

where $\boldsymbol{W}_{H} \in \mathbb{R}^{s \times 1}$ is a weight matrix. For the risk score prediction, SPIN computes a posterior probability as a risk score as follows: 


(4) \begin{align*}& \boldsymbol{Z}=\mathit{sigmoid}(\boldsymbol{W}_{H} \boldsymbol{H} + \boldsymbol{b}_{H}),\end{align*}

where $\boldsymbol{b}_{H}$ is a bias vector, and the risk score prediction is computed with a sigmoid activation function $\sigma = \frac{1}{1 + e^{-\boldsymbol{x}}}$. The output layer is fully connected with the last hidden layer.

## RESULTS

### SPIN improves the predictive performance compared with existing benchmark models

We assessed SPIN’s predictive performances in comparison with current sex-combined and -specific analysis benchmark models for the survival analysis and risk score prediction. In the survival analysis, we considered several cancers, indicating the top-four rates of difference in the incidence of men and women, which include liver, stomach, lung and brain cancers [4]. We used the Cancer Genome Atlas (TCGA) gene expression datasets (i.e. RNA-Seq) for those cancers: liver (LIHC), stomach (STAD), lung (LUAD/LUSC) and brain (GBM and LGG). The detail of datasets is provided in the supplementary document (S2. Datasets). GBM and LGG datasets were combined into a dataset as pan-glioma (GBM/LGG). We split each TCGA dataset into model development (80%) and testing (20%) datasets with stratified random sampling based on sex. The development dataset was further split into training (80%) and validation (20%) datasets. Then, the data normalization on each experiment was performed, and specifically, validation and testing sets were scaled with the mean and standard deviation obtained from the training set. Benchmarks included sex-combined and -specific analysis models. For the sex-combined analysis models, we used elastic net (Cox-EN), neural network (Cox-NN), Cox-PASNet [12] coupled with Cox Proportional Hazards regression models (Cox-PH), DeepHisCoM [14] and CNN-Cox [15]. For the sex-specific analysis model, Cox-EN was trained separately for male and female groups (sex-specific Cox-EN). The predictive performance for survival analysis was calculated using the Concordance index (C-index), a non-parametric statistic that evaluates concordance between actual survival and the prediction. These experiments were repeated 10 times for the reproducibility of the model performance. The details of the model training and the hyper-parameter optimization are described in the supplementary document (S3. Model training and evaluation).

**Figure 2:**
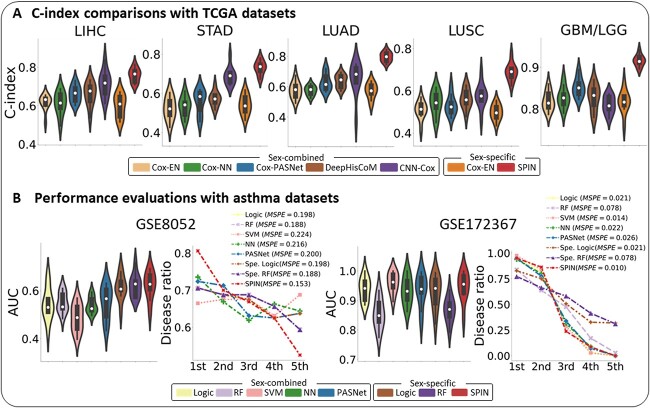
Performance evaluation for SPIN and other benchmark models with the TCGA and asthma datasets. (A) C-index comparison between SPIN and other benchmark models for the survival analysis. SPIN (red) outperforms other benchmarks across cancer datasets. (B) The graphical illustration of the performance comparisons with GSE8052 and GSE172367. For each dataset, we visualized the plots of the AUC (left) and disease ratio (right). In the disease ratio plot, the first group represents the highest risk group, whereas the fifth group represents the lowest risk group.

In the experiment, SPIN significantly outperformed all other benchmarks across the TCGA datasets (Figure 2A and Supplementary Table 1). SPIN yielded the C-index of $0.76 \pm 0.03$ in LIHC, $0.73 \pm 0.03$ in STAD, $0.80 \pm 0.03$ in LUAD, $0.69 \pm 0.04$ in LUSC and $0.92 \pm 0.01$ in GBM/LGG. These results demonstrate that SPIN achieved a remarkable improvement of C-index by an average of 9.7% for LIHC, 6.1% for STAD, 23.6% for LUAD, 19.6% for LUSC and 7.5% for GBM/LGG, compared with the performances of the second best benchmark model. The outperformance of SPIN to the second best benchmark was statistically validated by the Wilcoxon rank-sum test of C-index scores ($p < 5 \times 10^{-2}$) with all cancer datasets other than LIHC. We observed that Cox-EN, as either a sex-combined or -specific analysis model, produced the lowest C-index scores in all of the cancer datasets. The result implies that gene expression profiles of the cancer data are nonlinearly intertwined such that nonlinear-based models could be suitable for survival analysis.

Furthermore, we verified the robustness and reproducibility of SPIN using an additional external dataset. We considered a publicly available external dataset for cancer survival analysis, including RNA-seq and clinical information of sex, survival time and survival status in the Singapore Oncology Data Portal (OncoSG) [16]. The OncoSG dataset includes 169 RNA-seq data of east Asian patients with lung adenocarcinoma. We applied SPIN and the other benchmark models, which were trained with the TCGA LUAD dataset, to the OncoSG dataset. The performance was shown as similar with the results in the experiments with TCGA LUAD. The C-index of SPIN ($0.80 \pm 0.01$) was still significantly the highest compared with the benchmark, DeepHisCoM ($0.62 \pm 0.02$), Cox-PASNet ($0.54 \pm 0.03$), Cox-NN ($0.54 \pm 0.05$), Cox-EN ($0.54 \pm 0.03$), Sex-specific Cox-EN ($0.53 \pm 0.05$) and CNN-Cox ($0.48 \pm 0.07$) (Supplementary Table 2).

For risk prediction, we evaluated our SPIN framework using asthma datasets. Two publicly available asthma datasets were downloaded from the Gene Expression Omnibus (GEO) database (Accession ID: GSE8052 and GSE172367). Similarly, we stratified the asthma datasets based on sex and disease status (control/asthma) and normalized the datasets. For benchmarks, we used sex-combined analysis methods, including logistic regression (Logic), support vector machine (SVM), neural network (NN) and a pathway-associated sparse neural network (PASNet) [11]. For the sex-specific analysis, logistic regression (sex-specific Logic) and random forest (sex-specific RF) models were applied to separately train for males and females. The area under the receiver operating characteristic (ROC) curve (AUC) and disease ratios were computed to evaluate the performance for risk score prediction. We repeated these experiments 10 times.

In the experimental results with asthma datasets, SPIN produced the AUC of $0.62 \pm 0.05$ for GSE8052 and $0.95 \pm 0.05$ for GSE172367 (Figure 2B and Supplementary Table 3), which is competitive performance with SVM with the linear kernel. The competitive performance of GSE172367 is mainly due to the small data size (N=190), and the SPIN’s predictive performance will be empowered with larger training samples. Moreover, the distinct predictive performances between GSE8052 (AUC=0.62) and GSE172367 (AUC=0.95) were shown, since GSE172367 is from primary or target tissue (airway epithelium cells) for asthma. The target tissue/cell types have a well-known role in asthma pathogenesis and remodeling [17], whereas GSE8052 is from surrogate tissues (peripheral blood lymphocytes), which may not truly reflect the disease pathogenesis.

Furthermore, we assessed the stratification of risk scores by disease ratios with patient groups of similar severity. To stratify the patients, the test dataset was sorted by the predicted risk scores and divided into five groups. Each group estimated the disease ratio of the actual asthma cases to the total group populations. The disease ratios of each model are depicted in Figure 2B, where the first (or last) group reflects the highest (or lowest) risk group. Then, we computed the mean squared prediction error (MSPE) between the ideal and predicted disease ratios on the five groups. The ideal disease ratios were calculated by counting the total number of actual asthma cases to the five groups (e.g. number of actual asthma/number of a group populations) in consecutive order. The ideal disease ratios of GSE8052 are 1.0 (17/17), 1.0 (16/16), 1.0 (16/16), 0.31 (5/16) and 0.0 (0/16) in each group. SPIN obtained the lowest MSPE of 0.15 and 0.01 in GSE8052 and GSE172367, respectively, which reduced the error by 18.8% and 23.7% compared with the second lowest. Through this assessment of the disease ratio, SPIN showed its enhanced power to linearly stratify patients, as a risk score tool, compared with other benchmark models.

### SPIN identifies statistically significant sex-specific and -shared genes and pathways

Our global interpretation analysis reveals significant sex-specific and -shared biomarkers (genes/pathways) nonlinearly associated with clinical outcomes at a population level. Sex-specific importance scores of each gene/pathway are computed to approximate their relative importance on the predictive mechanisms. We determine statistically significant sex-shared factors if the results of statistical tests are significant in both sexes, and sex-specific factors if the statistical significance is indicated with only one between the sex groups ($P < 10^{-5}$ after FDR correction). The detailed algorithm is provided in S4; global interpretation analysis in the supplementary document. For the sake of simplicity, our global interpretation analysis was conducted with the TCGA data (GBM/LGG) for survival analysis and the asthma data (GSE172367) for risk score prediction using the optimal model that yielded the best predictive performance in Results.

**Figure 3:**
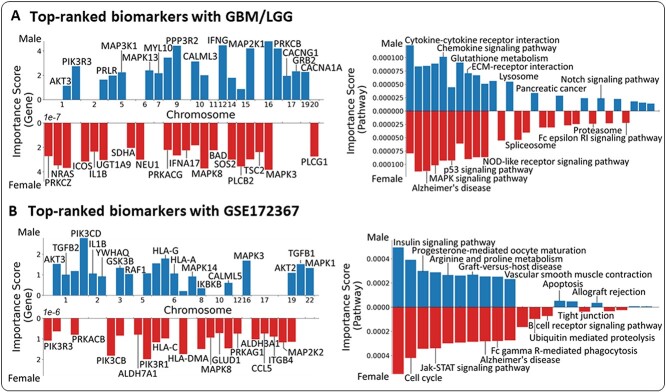
The barplot visualizations of the top-ranked genes (left) and biological pathways (right) (A) The most significant sex-specific and -shared biological risk factors from male (blue bars on upper side) and female (red bars on lower side) groups with GBM/LGG. (B) with GSE172367. In both (A) and (B), the bars shown in only one of the sex groups represent the sex-specific factors, whereas those shown in both male and female groups represent the sex-shared factors.

In GBM/LGG, we identified 2923 sex-shared, 502 male-specific and 704 female-specific genes as significant factors (Supplementary Figure 1). Among them, we explored the 10 top-ranked genes from each group (i.e. sex-shared, male-specific and female-specific groups), based on their highest importance scores. The top-ranked genes are illustrated and listed with their chromosome numbers, importance scores, *P*-values and related literature (Figure 3A (left) and Supplementary Table 4). For instance, *MAPK8* and *AKT3* appeared as sex-shared genes; *MAP3K1* and *IFNG* were shown as significant only in males, whereas *NRAS*, *PLCG1* and *TSC2* were significant only in females. The highly ranked genes are mostly reported as well-known biomarkers of pan-glioma in the biological literature. For instance, *MAPK8* [18] and *AKT3* [19] appeared as sex-shared genes; *MAP3K1* [20] and *IFNG* [21] were shown as significant only in males, whereas *NRAS* [22], *PLCG1* [23] and *TSC2* [24] were significant only in females. We also identified significant sex-specific and -shared biological pathways in GBM/LGG. We discovered 146 pathways enriched in both males and females, 11 significant pathways enriched in males and 15 pathways enriched in females. The top-10 pathways, ranked by their importance scores on each group, are shown in Figure 3A (right), such as *MAPK signaling pathway* [25] and *p53 signaling pathway* [26] as the sex-shared pathways; *Notch signaling pathway* [27] as a male-enriched pathway; and *Spliceosome* [28] as a pathway enriched in females (Supplementary Table 5).

In the asthma data, SPIN identified 1504 sex-shared, 423 male-specific and 282 female-specific genes (Supplementary Figure 2). Figure 3B (left) visualizes the 10 top-ranked genes of sex-shared, male-specific and female-specific groups. Sex-shared genes include *PIK3R1* [29], *HLA-G* [30, 31] and *IKBKB* [32]. Male-specific genes include *TGFB1* [33, 34], *MAPK1* [35] and *IL1B* [36, 37]; on the other hand, female-specific genes include *ALDH3A1* [38] and *ITGB4* [39] (Supplementary Table 6). For the pathway analysis with the asthma data, we identified 132 sex-shared, 5 male-enriched and 6 female-enriched pathways. Top-ranked sex-shared and -specific pathways are listed with the related literature, including *JAK-STAT signaling pathway* [40, 41] and *Arginine and proline metabolism pathway* [42] as the sex-shared pathways; *Apoptosis* [43] as a pathway enriched in males; and *Ubiquitin mediated proteolysis* [44, 45] as a female-enriched pathway (Figure 3B [right] and Supplementary Table 7).

Interestingly, conventional linear-based Cox-PH and statistical logistic regression models identified no genes ($P < 10^{-2}$ after FDR correction over all genes) as statistically significant for the gene-sex interaction (Material and Methods, Supplementary Table 4 and 6). This result implies that SPIN can identify biologically significant sex-specific and -shared genes that could be missed in conventional methods.

### SPIN provides an insight into the understanding of individual level biological process

Our local interpretation analysis explains pathway-based predictive processes at an individual level compared with the global interpretation analysis that identifies general biomarkers of the whole population (S5. Local interpretation analysis). Through the local interpretation, we (1) unveil individual processes of the biological pathways that have positive/negative impacts on a prediction, (2) identify discriminative mechanisms on subgroups of interest by extending the sample-based local interpretation, (3) analyze the predictive process of individual mechanisms on samples of interest for reliable prediction and (4) explore sexual dimorphism in the predictive mechanisms of individuals. The pathway-based local interpretation analysis identifies biological functions involved in a target biological system in a robust manner rather than gene-based interpretation. In this study, for simplicity, we focused on an asthma dataset (GSE172367) that produced the highest predictive performances, as the importance of the local features is explained with respect to the model prediction.

**Figure 4:**
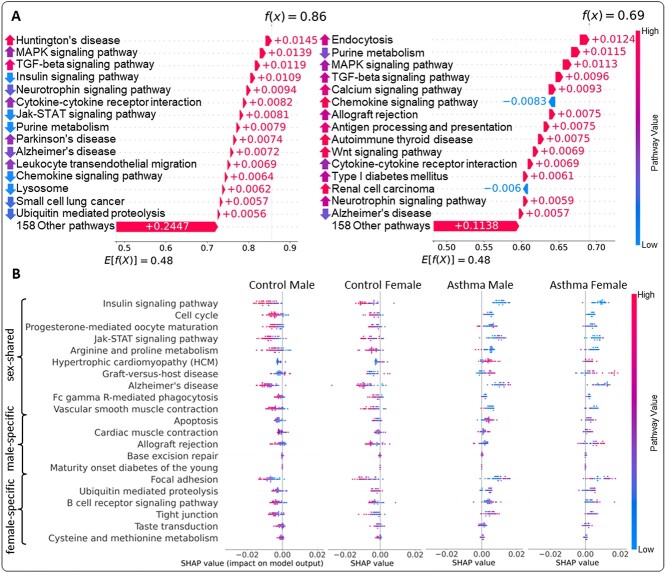
(A) SHAP waterfall plots of two individuals in asthma females. In the SHAP waterfall plots, the top-ranked 15 pathways of the patients, as well as the aggregate of SHAP values for 158 other pathways, are listed in descending order of the absolute SHAP values. For each local explanation, the base value at the bottom represents the expected value of the model output over the training dataset, and all SHAP values are summed up to the prediction. The relative enrichment of the pathways depicts the upward (enrichment) and downward (depletion) arrows. (B) SHAP summary plots of the top-ranked significant pathways from sex-shared, male-specific, and female-specific groups. An individual sample was represented by each dot on the visualization colored by its relative enrichment of the biological pathways. The data points were horizontally distributed based on their SHAP values.

First, we examined the pathway-based predictive processes of the asthma patients individually. The pathway effects on each individual prediction were estimated using the shapley additive explanations (SHAP) [46]. The SHAP explanation model assigns SHAP values to reflect the magnitudes and directions of the pathway effects on the prediction produced by SPIN. Then, the SHAP values and the relationships with the pathways’ enrichment were analyzed for the local interpretation. For instance, the local interpretations for two female patients with asthma are shown in Figure 4A. In the SHAP waterfall plots of Figure 4A, the top-ranked 15 pathways of the patients, as well as the aggregate of SHAP values for 158 other pathways, are listed in descending order of the absolute SHAP values. The SHAP waterfall plots illustrate how an individual risk score is computed from the inferred pathway values in a linear manner. The sum of the SHAP values of the pathways from a base value is equivalent to the risk score prediction: $f(x)=E[f(x)]+\sum \limits _{i} \phi _{i}$, where $f(x)$ is the risk score of given gene expression data $(x)$, $E[f(x)]$ is an expected value of the predictions for the other samples (i.e. the base value) and $\phi _{i}$ is the SHAP value for the $i$-th pathway. For the first patient on the left side in Figure 4A, the directions of the pathway effects on the risk score prediction ($f(x) = 0.86$) were all positive, indicating that the pathways of the patient are likely to increase the risk of asthma. Specifically, the local analysis shows that the enrichment of *Huntington’s disease* increases asthma risk by $+0.0145$ on the patient. On the other hand, the depletion of *Chemokine signaling pathway* increases the risk of asthma by +0.0064, which may imply that the enrichment of *Chemokine signaling pathway* is essential to control the asthma risk. Another female patient with asthma (Figure 4A [right]) shows that the enrichment of *Chemokine signaling pathway*, in contrast, results in a negative SHAP value ($\phi _{i} = -0.0083$), which decreases the asthma risk. This finding is aligned with the literature in which the dysfunction in *Chemokine signaling pathway* is correlated with the asthma severity [47]. Another negative SHAP value of *Renal cell carcinoma* along with *Chemokine signaling pathway* in the second patient also reduces the asthma risk score ($f(x) = 0.69$).

Secondly, we extended the individual local interpretation analysis to subgroups of interest to explore their broad distinctions of the pathway effects. We categorized the individuals into four groups: Control male, Control female, Asthma male and Asthma female. To determine what/how pathways cause differences between the subgroups, we analyzed the SHAP summary plots of the four subgroups, mainly considering the top-ranked sex-shared and -specific pathways identified in our global interpretation analysis (Figure 4B). The summary plots visualize the distribution on the individuals’ SHAP values in the four subgroups with their pathway values. The individual pathway values are colored in ranging between red (enriched) and blue (depleted). For instance, the SHAP values of *Insulin signaling pathway* (a sex-shared pathway) appeared negative in most males and females of the control group, whereas the asthma group showed positive values. The control group relatively exhibited high pathway values (enriched), but low pathway values in the asthma group, which implies that the enrichment of *Insulin signaling pathway* reduces the susceptibility to asthma, aligned with the literature [48]. By contrast, it is shown that the enrichment of another sex-shared pathway, *Hypertrophic cardiomyopathy (HCM)*, causes the development of asthma, but the depletion of the pathway has negative impacts on the risk score prediction. Furthermore, the male-enriched pathway, *Apoptosis*, is enriched in males more than females, which may increase asthma risk.

**Figure 5:**
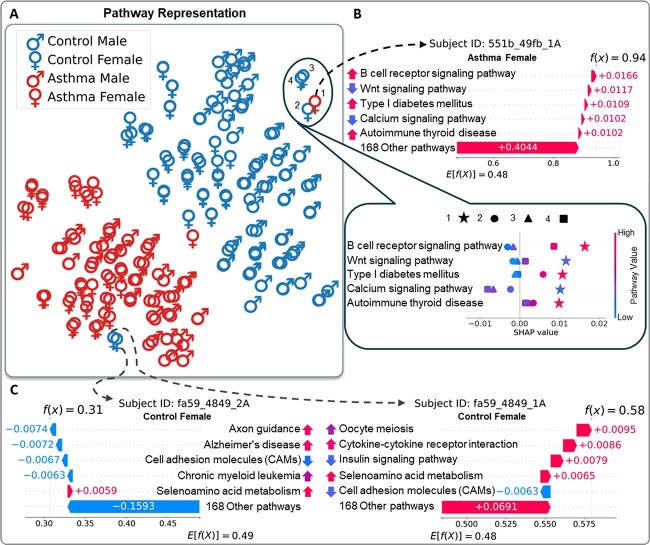
(A) The t-SNE plot of SPIN’s sex-specific pathway layers colored by asthma status (control/asthma). Each sample is with a male or female symbol. (B) The SHAP waterfall plot and summary plot for the numbered samples (1, 2, 3 and 4) in the circle of the top-right corner in Figure 5A. (C) The SHAP water plot of two control females. In the SHAP waterfall plots, the upward (enrichment) and downward (depletion) arrows represent the relative enrichment of the pathways.

Thirdly, we further investigated the predictive process of individual samples of interest to provide the reliability for the SPIN’s predictions. We focused on three individuals whose clinical outcomes are opposite to the adjacent samples, which are presumably outliers (Figure 5A). The subject ID of 551b_49fb_1A (Number 1 in the circle on the top-right corner of Figure 5A) is a female patient of the asthma group mostly neighboring the control females (Numbers 2, 3 and 4). Most pathways in the SHAP local explanation of 551b_49fb_1A, including *B cell receptor signaling pathway* ($\phi _{i} = +0.0166$), *Wnt signaling pathway* ($\phi _{i} = +0.0117$), *Type I diabetes mellitus* ($\phi _{i} = +0.0109$), *Calcium signaling pathway* ($\phi _{i} = +0.0102$) and *Autoimmune thyroid disease* ($\phi _{i} = +0.0102$), are associated with the susceptibility of asthma, which results in SPIN’s high risk score prediction ($f(x) = 0.94$) (the top of Figure 5B, the SHAP waterfall plot). Specifically, the enrichment of *B cell receptor signaling pathway*, *Type I diabetes mellitus* and *Autoimmune thyroid disease* in 551b_49fb_1A are associated with the risk of asthma. The depletion of *Wnt signaling pathway* and *Calcium signaling pathway* in the 551b_49fb_1A shows the high impact on the risk score. Our analysis shows that the effects of the pathways in 551b_49fb_1A (Number 1 and star symbol) reflect higher impacts on the risk score than the other control individuals (the bottom of Figure 5B, the SHAP summary plot), although the samples neighbor each other in the t-SNE plot, whereas the other three control females show negative effects on most pathways, which leads to the low risk score predictions ($f(x) = 0.20$, $0.32$ and $0.27$). B cell receptor signaling pathway demonstrated depletion in the two control females (Number 2 with circle symbol and Number 3 with triangle symbol), indicating a negative impact on their risk scores. *Calcium signaling pathway* in three control females mitigates the asthma risk, and the depletion of *Autoimmune thyroid disease* in the other three control individuals leads to the lower impacts on the risk scores than in 551b_49fb_1A.

Similarly, we explored two females of the control group, fa59_4849_2A and fa59_4849_1A, adjacent to the female patients of the asthma group. In the SHAP local explanation of fa59_4849_2A, most pathways, including *Axon guidance* ($\phi _{i} = -0.0074$), *Alzheimer’s disease* ($\phi _{i} = -0.0072$), *Cell adhesion molecules (CAMs)* ($\phi _{i} = -0.0067$) and *Chronic myeloid leukemia* ($\phi _{i} = -0.0063$), alleviate the risk of asthma, contributing to the low risk score ($f(x) = 0.31$) (the left SHAP waterfall plot of Figure 5C). However, most pathways of fa59_4849_1A show susceptibility to asthma (e.g. *Oocyte meiosis* ($\phi _{i} = +0.0095$), *Cytokine–cytokine receptor interaction* ($\phi _{i} = +0.0086$), *Insulin signaling pathway* ($\phi _{i} = +0.0079$) and *Selenoamino acid metabolism* ($\phi _{i} = +0.0065$)), which cause the risk score to be on the borderline ($f(x) = 0.58$) (the right SHAP waterfall plot of Figure 5C). It may imply that the subject of fa59_4849_1A has a high chance to develop asthma, although she is currently in asthma control.

**Figure 6:**
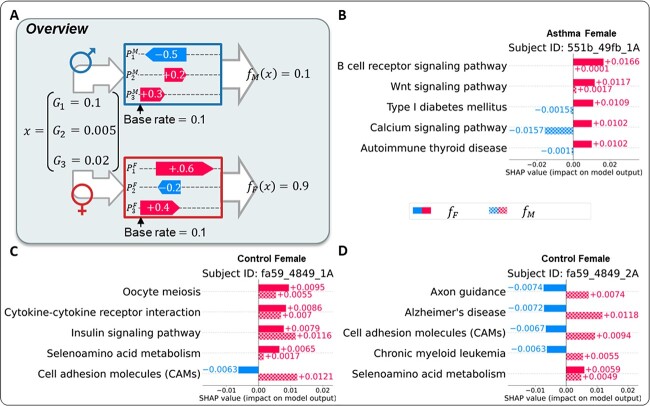
(A) The overview of the comparison of the pathway-based predictive mechanisms between males and females. We denote the SHAP explanation model and its estimated SHAP value as $f_{M}(.)$ and $\phi ^{f_{M}}$ for males and $f_{F}(.)$ and $\phi ^{f_{F}}$ for females, respectively. (B-D) SHAP cohort bar plots of the previous female samples depicted in Figure 5. For the pathways in each plot, their effects of an original (female) and the opposite (male) mechanisms represent the solid (upper) and hatched (lower) colors, respectively.

Lastly, we analyzed how sexual dimorphism affects an individual’s predictive mechanism. In particular, we compared the effects of sex-specific pathways contributing to the risk score predictions, as SPIN generates the sex-specific pathway representations of a given gene expression profile depending on the sex. We denote the SHAP explanation model and its estimated SHAP value as $f_{M}(.)$ and $\phi ^{f_{M}}$ for males and $f_{F}(.)$ and $\phi ^{f_{F}}$ for females, respectively. Figure 6A illustrates an example of the SPIN’s interpretation processes depending on sex. In the example, the sex-specific pathway effects result in the different risk score predictions ($f_{M}(x) = 0.1$, $f_{F}(x) = 0.9$). In particular, the *Calcium signaling pathway* of 551b_49fb_1A (asthma female) produces a high-positive effect ($\phi ^{f_{F}} = +0.0102$) to increase risk scores on the female-specific process, but it shows a high-negative effect ($\phi ^{f_{M}} = -0.0157$) if the individual is male with the same gene expression profile (Figure 6B). The effect of the *B cell receptor signaling pathway*, a significant female-enriched pathway in the previous summary plot of Figure 4B, presents a relatively high impact in females but a low impact in males. Furthermore, *CAMs* in both control female samples (fa59_4849_1A and fa59_4849_2A) have negative impacts on their risk scores (fa59_4849_1A: $\phi ^{f_{F}} = -0.0063$, fa59_4849_2A: $\phi ^{f_{F}} = -0.0067$), whereas the pathway effect on the male-specific process shows a positive impact on the prediction (fa59_4849_1A: $\phi ^{f_{M}} = +0.0121$, fa59_4849_2A: $\phi ^{f_{M}} = +0.0094$) (Figure 6C, D). Not only do *Calcium signaling pathway* and *CAMs* have an opposite direction between the male and female mechanisms, but other pathways (e.g. *Type I diabetes mellitus*, *Autoimmune thyroid disease*, *Axon guidance*, *Alzheimer’s disease*, *Chronic myeloid leukemia*) also demonstrate such sex disparities. These findings indicate that the biological mechanisms between males and females are distinct, suggesting the net canceling effects that particularly have the opposite directions between sexes occurs in the sex-combined analysis frameworks.

## DISCUSSION

In this study, we introduced SPIN, a novel unified biologically interpretable DL framework for sexual dimorphism analysis. SPIN predicts sexual dimorphic outcomes of the disease with the gene expression profiles of males and females simultaneously and offers advanced interpretability with statistical significance tests by incorporating prior biological knowledge. As a result, SPIN outperformed other sex-combined or sex-specific benchmark models across several publicly available cancer datasets. Moreover, SPIN captures complex and nonlinear hierarchical feature representations which are often missed by existing approaches. By leveraging the complex relationships in SPIN with sexual dimorphic data, we not only identify statistically significant sex-specific and -shared risk factors (i.e. genes/pathways) at a population level, but also analyze how the biological pathways lead to predictions at an individual level. To the best of our knowledge, SPIN is the first unified DL framework for sexual dimorphism analysis to discover potential sex-specific/-shared biomarkers in complex human diseases.

SPIN is biologically interpretable, inherently relying on pathway databases for the architecture design. The sparse connections between genes and pathway layers in SPIN are constrained by biological pathways, which consequently make the model dependent on the quality of the annotations. Incorporating multiple pathway databases (e.g. Reactome) or ontologies (e.g. GO) will provide robust analyses without bias to a specific database. Moreover, SPIN’s pathway-based architecture design allows only genes belonging to the pathways in the model, which excludes a number of genes that have not been annotated for pathways. However, rapid advancement and development of larger pathway databases will include more genes in SPIN for the pathway-based analysis.

SPIN could provide potential novel sex-specific biomarkers for prognosis and genetic susceptibility in complex human diseases. We validated several statistically significant sex-shared genes/pathways. For example, *MAPK8* [18], *AKT3* [19], *MAPK signaling pathway* [25] and *p53 signaling pathway* [26] are known biomarkers in brain tumors, while *PIK3R1* [29], *HLA-G* [31], *IKBKB* [32], *JAK-STAT signaling pathway* [40] and *Arginine and proline metabolism pathway* [42] are known for asthma. Although we identified sex-specific genes/pathways, we acknowledge that there is limited sexual dimorphism related literature, so we cannot validate all our findings.

Altogether, we showed that DL approaches applied to sexual dimorphism complex disease are highly accurate at predicting sex-specific and shared risk loci and pathways, providing proof of concept that this approach may lead to a mechanistic understanding of a sex differences precision medicine approach.

**Key Points**

- SPIN is a general unified framework that analyzes sexual dimorphism using omics data with multiple applications.
- SPIN improves predictive power compared with existing sex-combined/-specific analysis models.
- SPIN identifies sex-specific and -shared genes and pathways nonlinearly associated with clinical outcomes.
- SPIN characterizes biological processes on each individual sample, leading to the development of precision medicine tailored to a specific individual’s characteristics.

## Supplementary Material

Supplementary_bbae239

## Author Biographies

**Euiseong Ko** is a PhD student in the Department of Computer Science at the University of Nevada, Las Vegas. His research interest is Machine Learning and Deep Learning in Bioinformatics and Healthcare.

**Youngsoon Kim** is currently an assistant professor in the Department of Information and Statistics and Department of Bio & Medical Bigdata (BK21 Four program), Gyeongsang National University, South Korea. She received her BS, MS, and PhD degrees in statistics from Gyeongsang National University, South Korea, in 1994, 1998, and 2005, respectively. From 2009 to 2020, she was a senior researcher at the GyeongNam Institute, South Korea. She was a postdoctoral researcher and part-time assistant professor at Kennesaw States University from 2017 to 2019. Her research interests include bioinformatics, machine learning, data mining, and big data analytics.

**Farhad Shokoohi** is currently an assistant professor of Statistics in the Department of Mathematical Sciences at the University of Nevada, Las Vegas. He earned his PhD in Statistics from Shahid Beheshti University in Tehran, Iran, in 2012. Before his current position, he held several academic roles, including Lecturer at McGill University, Canada (2018–2019), assistant professor at the Concordia University, Canada (2017–2018), Postdoctoral Fellow at the McGill University (2013–2017), Visiting Scholar at the Ohio State University, USA (2014), and Postdoctoral Fellow at the University of Manitoba, Canada (2013). His research interests encompass Statistics, Data Science, Machine Learning, Statistical Genetics and Genomics, and High-dimensional Data Analysis.

**Tesfaye Mersha** is a Professor of Pediatrics in the Department of Pediatrics, Cincinnati Children's Hospital Medical Center, and University of Cincinnati College of Medicine, where he leads the Population Genetics, Ancestry and Bioinformatics Laboratory (pGAB). His research combines genetic ancestry, bioinformatics, and statistical and functional genomics to unravel genetic and non-genetic contributions to complex diseases in human populations, particularly in allergic disorders. Much of his research is at the intersection of basic, clinical, and translational research, and he is interested in crossline disciplines to dissect how biologic predisposition and socio-environmental exposures interact to shape racial disparities in asthma and other complex disorders. Dr. Mersha is recognized in the field of genetic ancestry, race, ethnicity, admixture mapping, and functional genomics related to complex diseases. His current research has focused on understanding the complex interplay surrounding environmental exposures, genomics, race, ethnicity, ancestry, multi-omics, and health, especially in under-represented populations. He is a strong advocate of reimagining health equity in the era of precision medicine, specifically addressing the lack of equity (or racial disparities) in genomic research through inclusion of diverse populations.

**Mingon Kang** is an assistant professor in the Department of Computer Science at the University of Nevada, Las Vegas. His research interests include Machine Learning, Big Data Analytics, Data Science, and Bioinformatics. Specifically, Dr. Kang has been focusing on developing novel computational methodologies for integrative and interpretable deep learning. Dr. Kang obtained his PhD and master degrees from the University of Texas at Arlington in 2015 and 2010 respectively, and he has a BE in Computer Engineering from the Hanyang University in South Korea in 2006.
