# Supplementary material for: SPIN: sex-specific and pathway-based interpretable neural network for sexual dimorphism analysis
Source: Brief Bioinform. 2024 May 28;25(4):bbae239. doi: 10.1093/bib/bbae239 (PMC11133003; doi:10.1093/bib/bbae239)
Supplement: Supplementary_bbae239 [file supplementary_bbae239.pdf]

SPIN: Sex-specific and Pathway-based Interpretable Neural Network for Sexual Dimorphism Analysis

Euiseong Ko[1], Youngsoon Kim[2], Farhad Shokoohi[3], Tesfaye B. Mersha[4*] and Mingon Kang[1*]

[1] Department of Computer Science, University of Nevada, Las Vegas, Las Vegas, NV 89154, USA

[2] Department of Information and Statistics and Department of Bio & Medical Bigdata (BK21 Four program), Gyeongsang National University, Jinju, Republic of Korea

[3] Department of Mathematical Sciences, University of Nevada, Las Vegas, Las Vegas, NV 89154, USA

[4] Department of Pediatrics, Cincinnati Children's Hospital Medical Center, University of Cincinnati, Cincinnati, OH, USA

[*] Corresponding author. tesfaye.mersha@cchmc.org, mingon.kang@unlv.edu

S1. Sparse coding

Our novel sparse coding prunes the neural network connections for model interpretability. The optimal binary mask matrix MP is constructed on each connection between layers. The mask matrix is generated by:

$$M_l = 1(|W_l| \geq S_l), \quad l \neq 1,$$

where S_l is the optimal sparsity level, and the function $1(x)$ returns either one or zero determined by the conditions of x. For feasible computation, we set a finite range of the sparsity level $S = [0, 100)$ that represents the ratio of weights to be zero-out. The sparsity level of zero means a fully-connected layer, whereas that of 100 means no connections. Then a cubic-spline interpolation is used to figure out the optimal sparsity level estimated to minimize the cost function with respect to sparsity levels.

S2. Datasets

In this study, we used gene expression profiles (i.e., RNA-Seq) from cancers and asthma for two case studies: (1) survival analysis and (2) risk score prediction. For survival analysis, TCGA datasets, including liver hepatocellular carcinoma (LIHC), stomach adenocarcinoma (STAD), lung adenocarcinoma (LUAD), lung squamous cell carcinoma (LUSC), and an integrated dataset of glioblastoma multiforme (GBM) and brain lower grade glioma (LGG), were obtained from cBioPortal (www.cbioportal.org/datasets). For risk score prediction, we obtained two asthma data from the Gene Expression Omnibus (GEO, https://www.ncbi.nlm.nih.gov/geo), including lymphoblastoid cell line (GSE8052) and airway epithelial cell (GSE172367). We excluded genes that are redundant or are missing values for over 80% of their samples. The KEGG pathway database was obtained from the Molecular Signature Database (MSigDB) for pathway-based analysis. We considered 173 pathways whose gene sizes are between 15 and 300, since small pathways are often redundant with other larger pathways, and large pathways are related to general biological pathways that are not specific to a certain disease. Accordingly, we considered genes belonging to the pathways as an input to the model. The details of datasets are provided in Supplementary Table 1 and 2.

S3. Model training and evaluation

The model parameters of SPIN are optimized by minimizing the objective function: the average negative log partial likelihood for survival analysis and the binary cross-entropy for risk score prediction. To tackle the major challenges, such as avoiding overfitting and reducing computational complexity of training on high-dimensional low sample size (HDLSS) data, sparse coding is applied to SPIN by trimming connections

that unaffected the minimization of the loss on each epoch until convergence. We also used the dropout and L^2 regularization. Additionally, we considered class weights on the objective function to avoid bias to the majority class in the imbalanced data. SPIN involves the following hyperparameters: (1) learning rate, (2) dropout rate, and (3) weight decay (L^2 penalty). We used ReLU as an activation, kaiming uniform (a.k.a. He initialization) as an initializer, and Adam as an optimizer. With the training and validation sets, the optimal hyperparameters are heuristically determined on each experiment using grid search. The optimal model is then applied to the testing set to assess the C-index and AUC for survival analysis and risk prediction, respectively.

S4. Global interpretation analysis

We compute importance scores of each gene/pathway by interpreting the optimized SPIN's parameters. An importance score on each gene/pathway reflects a magnitude how much a gene/pathway contributes to the predictive outcome. A partial derivative of a function $\frac{\partial f(x)}{\partial x}$ measures how the predictive function $f(x)$ changes as the given gene/pathway value (x) is changed. We define the importance score on each node in a layer by accumulating gradients along the path from x to $f(x)$. Specifically, the calculation procedures for partial derivatives on pathways and genes are as follows: For pathways,

$$\begin{aligned}\frac{\partial Z}{\partial P^M} &= \frac{\partial Z}{\partial H}\frac{\partial H}{\partial P^M W_P}\frac{\partial P^M W_P}{\partial P^M},\\ \frac{\partial Z}{\partial P^F} &= \frac{\partial Z}{\partial H}\frac{\partial H}{\partial P^F W_P}\frac{\partial P^F W_P}{\partial P^F},\end{aligned} \quad (1)$$

where W_P is the matrix of sparse connections between the sex-specific pathway layers and the hidden layer. For genes,

$$\begin{aligned}\frac{\partial Z}{\partial G^M} &= \frac{\partial Z}{\partial H}\frac{\partial H}{\partial P^M W_P}\frac{\partial P^M W_P}{\partial P^M}\frac{\partial P^M}{\partial G^M W_G^M}\frac{\partial G^M W_G^M}{\partial G^M},\\ \frac{\partial Z}{\partial G^F} &= \frac{\partial Z}{\partial H}\frac{\partial H}{\partial P^F W_P}\frac{\partial P^F W_P}{\partial P^F}\frac{\partial P^F}{\partial G^F W_G^F}\frac{\partial G^F W_G^F}{\partial G^F},\end{aligned} \quad (2)$$

where W_G^M, W_G^F are the matrices of connections regulated by the biological prior knowledge between genes and pathways. Supplementary Figure 3 shows an architecture diagram of SPIN in which each layer, weight, and gradient calculation are notated. For the global interpretable model, we applied the averaged optimal parameters (i.e., weights and nodes) of each optimized SPIN model of ten experiments to capture the robust significant biological factors. We generated a matrix reflecting the gene or pathway importance ($\in \mathbb{R}^{n\times q}$ or $\mathbb{R}^{n\times r}$).

The statistical significance of the importance score distributions for each gene/pathway is tested using the one-sample *t*-test under the null hypothesis of zero-mean ($H_0: \mu = 0$). We assume that the zero

value of the partial derivative for a gene/pathway means no impact on the SPIN prediction. The *t*-test of the importance score distributions is performed separately for male and female groups to prevent canceling effects that may happen when males and females act in opposite directions on specific genes/pathways. Then, the resulting p-values are corrected for multiple testing using a false-discovery rate (FDR)-controlling method (the *Benjamini-Hochberg* (BH) procedure) in which we set the family-wise error rate (FWER) as 10^{-2}.

We conducted the gene-sex interaction analysis using the conventional linear-based statistical method. For survival analysis, a regression Cox-PH model was used to fit gene expression, sex, and the interaction between them. For risk score prediction, we used a logistic regression model as a linear combination of gene expression, sex, and the interaction. To be specific,

$$type_{g,i} = \beta_0 + \beta_1 expr_{g,i} + \beta_2 sex_i + \beta_3 expr_{g,i} sex_i, \quad (3)$$

where $type_{g,i}$ is the target label for gene g and sample i, $expr_{g,i}$ is sample i's expression level for gene g, and sex_i is sample i's value for the sex. Based on this representation, we consider an interaction effect between a gene and sex in regard to asthma takes place if the learned β_3 coefficient is statistically significant (after FDR correction over all genes).

S5. Local interpretation analysis

Compared to the global interpretation analysis that identifies *'what'* significant features (i.e., genes/pathways) are involved in a biological system from the whole population, the local interpretation analysis scrutinizes each individual sample on *'how'* the features contribute to the target outcomes. Specifically, the local interpretation analysis reveals how the features have positive (or negative) impacts on a particular prediction using Shapley Additive Explanations (SHAP) based on a concept from game theory called the Shapley value [29]. SHAP assigns a value ϕ_i of the magnitude for the feature effect on the prediction p to a feature i as follows:

$$\phi_i(p) = \sum_{S \subseteq N \setminus i} \frac{|S|!(n-|S|-1)!}{n!} (p(S \cup i) - p(S)), \quad (4)$$

where $(p(S \cup i) - p(S))$ represents the difference in predictions when the feature i is included and excluded. $\frac{|S|!(n-|S|-1)!}{n!}$ represents the weighting for the marginal contributions. The total sum of all SHAP values is equal to the predicted probability of a sample (a.k.a. the local additivity property of SHAP), so that the SHAP value of each feature reflects the relative impact on the prediction.

In our local interpretation analysis, we utilized a SHAP explanation model (i.e., DeepExplainer) that approximates SHAP values for deep learning models, applying the intermediate layer (pathway layer in SPIN) into DeepExplainer to obtain SHAP values of the pathways. We note that we separately used the

DeepExplainer for males and females, since the architecture of SPIN contains male- and female-specific pathway layers. We observed various patterns of pathway contributions among individual samples, which cannot be identified by the global interpretation analysis. For instance, one of the widely known asthma-related pathways, *Jak-STAT signaling pathway* [14, 24], indicates high influence on the risk of asthma as shown in the global interpretation analysis (the right side in Fig. 3B). However, the second patient of asthma female in Fig. 4A shows that *Jak-STAT signaling pathway* might not have significant influence on risk of asthma. Instead, *Endocytosis* was detected as the most influential pathway for the patient.

Supplementary Figures

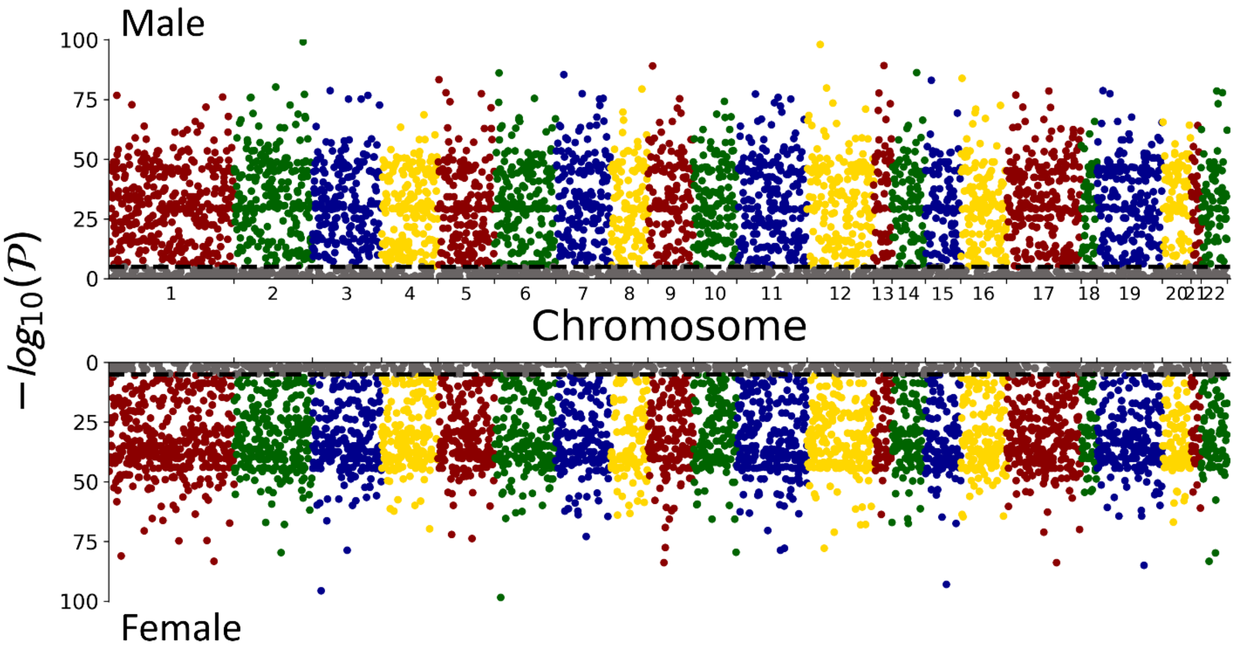

Supplementary Figure 1. Miami plot of GMB/LGG. Among the total number of 4350 genes in GBM/LGG, we identified 3425 genes for males and 3627 genes for females as the significant factors. In the plot, each point represents a gene belonging to the corresponding chromosomes shown in x-axis. The y-axis shows the adjusted p-values, where significant genes colored by red, green, blue, and yellow ($p < 10^{-5}$ after FDR correction across all genes), whereas insignificant genes colored by gray.

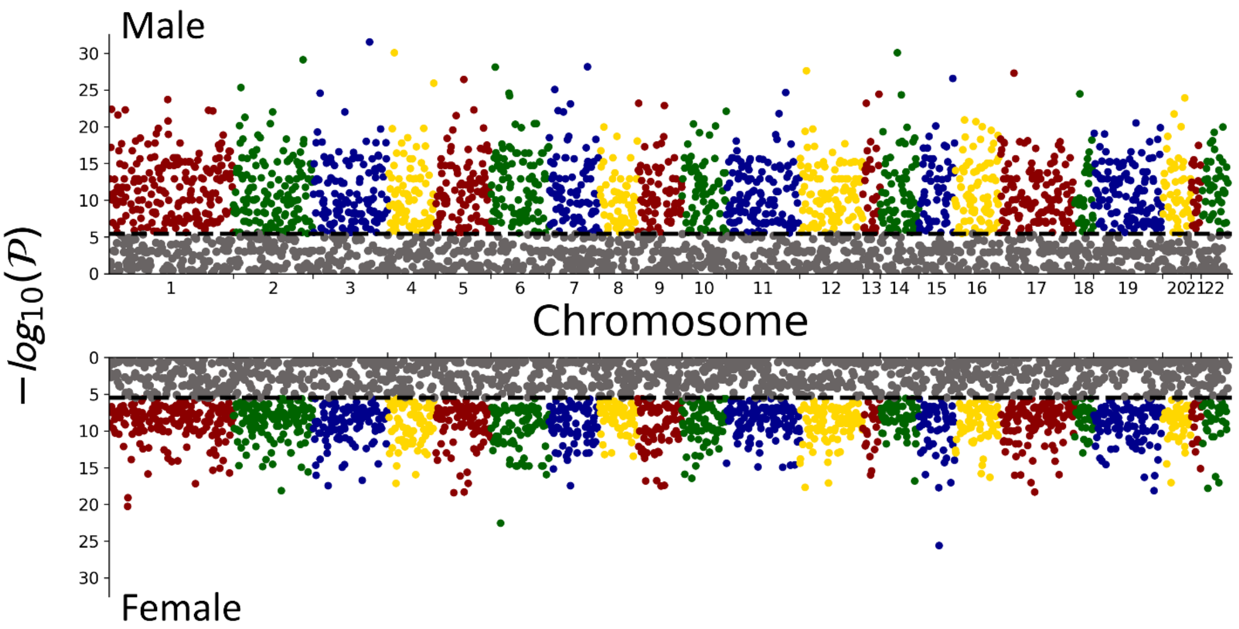

Supplementary Figure 2. Maimi plot of GSE172367. Among the total number of 3019 genes in GSE172367, 1927 genes for males and 1786 genes for females.

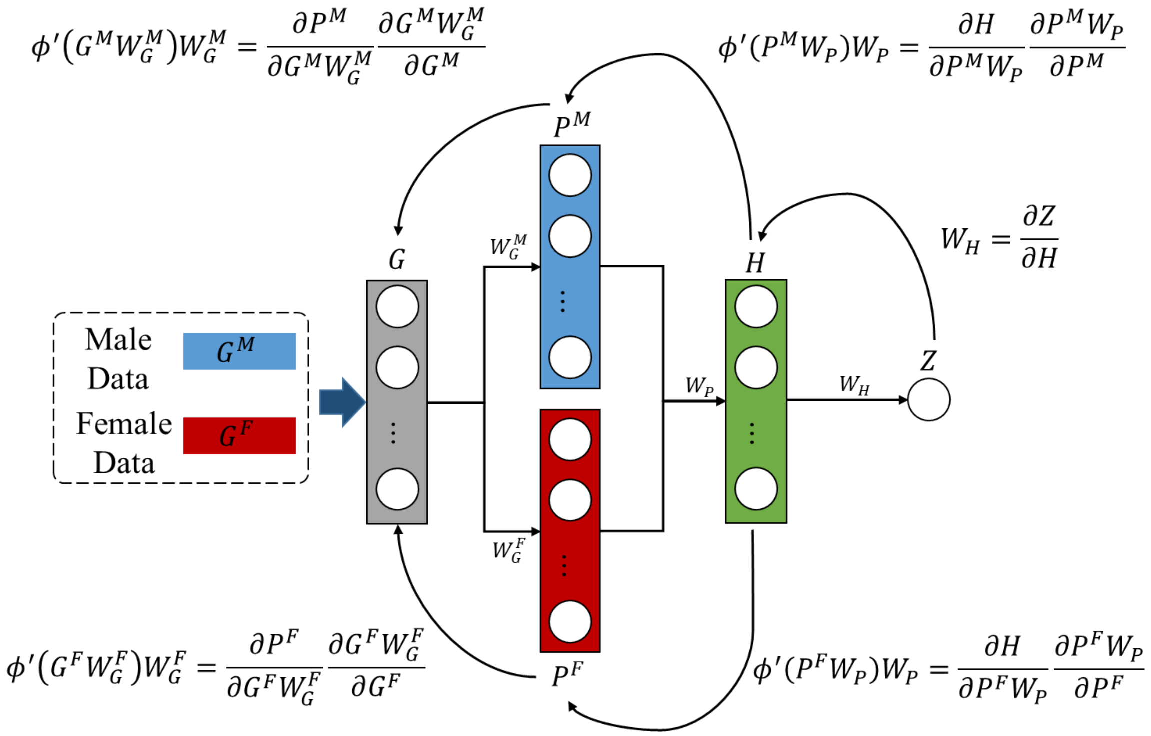

Supplementary Figure 3. The diagram of SPIN. For each layer and weight, M and F are denoted for male and female, respectively. G^M and G^F are gene expression data. W_G^M and W_G^F are the matrices of connections regulated by the biological prior knowledge between genes and pathways. P^M and P^F are the pathway layers. W_P is the matrix of sparse connections between the sex-specific pathway layers and the hidden layer. H is a hidden layer, and W_H is a weight matrix between hidden and output layers. Each ∂ stands for gradient calculation.

Supplementary Tables

Supplementary Table 1. The performance comparisons of TCGA datasets.

C-Index		Data				
Models		LIHC	STAD	LUAD	LUSC	GBM/LGG
Sex-combined	Cox-EN	.616 ± .038	.523 ± .054	.577 ± .051	.508 ± .040	.819 ± .022
	Cox-NN	.612 ± .056	.532 ± .047	.574 ± .029	.542 ± .053	.827 ± .024
	Cox-PASNet	.662 ± .041	.558 ± .057	.630 ± .053	.531 ± .044	.852 ± .022
	DeepHisCoM	.660 ± .056	.571 ± .036	.635 ± .039	.562 ± .044	.828 ± .028
	CNN-Cox	.691 ± .067	.689 ± .044	.643 ± .092	.576 ± .053	.810 ± .020
Sex-specific	Cox-EN	.599 ± .055	.541 ± .046	.572 ± .048	.498 ± .034	.817 ± .026
	SPIN	.758 ± .033	.731 ± .033	.795 ± .029	.689 ± .038	.916 ± .013

Supplementary Table 2. The predictive performance with TCGA LUAD and OncoSG LUAD datasets.

C-Index		Data	
Models		TCGA-LUAD	OncoSG-LUAD [30]
Sex-combined	Cox-EN	.577 ± .051	.535 ± .030
	Cox-NN	.574 ± .029	.541 ± .046
	Cox-PASNet	.630 ± .053	.536 ± .027
	DeepHisCoM	.635 ± .039	.621 ± .016
	CNN-Cox	.643 ± .092	.479 ± .073
Sex-specific	Cox-EN	.572 ± .048	.532 ± .053
	SPIN	.795 ± .029	.798 ± .014

Supplementary Table 3. The performance comparisons of asthma datasets.

AUC		Data	
Models		GSE8052	GSE172367
Sex-combined	Logistic	.544 ± .063	.939 ± .037
	RF	.550 ± .040	.854 ± .047
	SVM	.496 ± .060	.961 ± .033
	NN	.540 ± .031	.932 ± .044
	PASNet	.552 ± .065	.934 ± .052
Sex-specific	Logistic	.614 ± .049	.929 ± .052

**RF**	.620 ± .045	.868 ± .039
**SPIN**	.622 ± .054	.947 ± .046

Supplementary Table 4. The top-ranked genes of GBM/LGG

	Gene	Chr[1]	Male		Female		Score[2] (×1e-7)	Interaction *p*-value[3]
			p-value	Score (×1e-7)	*p*-value	Score (×1e-7)		
**Sex-shared**	MAPK3	16	< 0.00001	4.79	< 0.00001	3.84	4.79	0.965
	PPP3R2	9	< 0.00001	4.43	< 0.00001	2.66	4.43	0.793
	MAP2K1	15	< 0.00001	4.21	< 0.00001	2.98	4.21	0.994
	MAPK8	10	< 0.00001	2.00	< 0.00001	3.73	3.73	0.511
	AKT3	1	< 0.00001	1.14	< 0.00001	3.69	3.69	0.447
	PLCB2	15	< 0.00001	8.70	< 0.00001	3.57	3.57	0.913
	PRKACG	9	< 0.00001	3.45	< 0.00001	2.21	3.45	0.899
	CALML3	10	< 0.00001	3.13	< 0.00001	1.83	3.13	0.612
	UGT1A9	2	< 0.00001	1.64	< 0.00001	3.04	3.04	0.705
	SOS2	14	< 0.00001	1.79	< 0.00001	3.00	3.00	0.380
**Male-specific**	IFNG	12	< 0.00001	4.46	-	-	-	0.869
	PRKCB	16	< 0.00001	4.20	-	-	-	0.934
	PIK3R3	1	< 0.00001	2.74	-	-	-	0.915
	MAPK13	6	< 0.00001	2.40	-	-	-	0.713
	GRB2	17	< 0.00001	2.33	-	-	-	0.858
	CACNA1A	19	< 0.00001	2.27	-	-	-	0.894
	MAP3K1	5	< 0.00001	2.24	-	-	-	0.920
	MYL10	7	< 0.00001	2.17	-	-	-	0.555
	PRLR	5	< 0.00001	1.98	-	-	-	0.557
	CACNG1	17	< 0.00001	1.95	-	-	-	0.934
**Female-specific**	NRAS	1	-	-	< 0.00001	3.48	-	0.811
	ICOS	2	-	-	< 0.00001	3.16	-	0.980
	NEU1	6	-	-	< 0.00001	3.01	-	0.800
	PLCG1	20	-	-	< 0.00001	2.75	-	0.925
	PRKCZ	1	-	-	< 0.00001	2.72	-	0.748
	TSC2	16	-	-	< 0.00001	2.39	-	0.999
	IL1B	2	-	-	< 0.00001	2.35	-	0.524
	IFNA17	9	-	-	< 0.00001	2.24	-	0.586
	BAD	11	-	-	< 0.00001	2.24	-	0.644

SDHA	5	-	-	< 0.00001	2.05	-	0.930

[1]Chr is the corresponding chromosomes. [2]Score represents a bigger *importance score* between male and female by our global interpretation. [3]Interaction *p*-value indicates the *p*-value of interaction variables in the conventional statistical approach.

In the sex-shared genes, *MAPK8*, an autophagy-related gene, is a protective factor for GBM survival [20]. *AKT3* has an influence on tumor suppressive function in GBM and activates DNA repair and resistance to radiation and chemotherapy in GBM [22]. For the male-specific genes, *MAP3K1* is highly expressed in peripheral infiltrating GBM cells than in normal tissues [27]. *IFNG* contributes to the anti-tumor immune response in the GBM microenvironment [11]. As the female-specific genes, *NRAS* is upregulated in the glioma pathways [23]. High expression of *PLCG1* is relevant to tumor progression and poor survival in LGG. Additionally, *PLCG1* is reported to exert influence on the growth, migration, and invasiveness of LGG cells [13]. The mutation of *TSC2* found in peripheral blood is recognized in glioblastoma as well as in glioblastoma-derived cells [19].

Supplementary Table 5. The top-ranked pathways of GBM/LGG

	**Pathway**	**Male**		**Female**		**Score** (×1e-4)
		p	**Score** (×1e-4)	***p***	**Score** (×1e-4)	
**Sex-shared**	Cytokine-cytokine receptor interaction	< 0.00001	1.23	< 0.00001	0.79	1.23
	Complement and coagulation cascades	< 0.00001	0.84	< 0.00001	1.13	1.13
	Alzheimer's disease	< 0.00001	0.85	< 0.00001	1.12	1.12
	MAPK signaling pathway	< 0.00001	0.89	< 0.00001	1.02	1.02
	Chemokine signaling pathway	< 0.00001	1.02	< 0.00001	0.93	1.02
	p53 signaling pathway	< 0.00001	0.45	< 0.00001	0.93	0.93
	Glutathione metabolism	< 0.00001	0.91	< 0.00001	0.61	0.91
	ECM-receptor interaction	< 0.00001	0.71	< 0.00001	0.9	0.9
	Leishmania infection	< 0.00001	0.67	< 0.00001	0.87	0.87
	NOD-like receptor signaling pathway	< 0.00001	0.51	< 0.00001	0.87	0.87
**Male-specific**	Nucleotide excision repair	< 0.00001	0.56	-	-	-
	Lysosome	< 0.00001	0.55	-	-	-
	Pancreatic cancer	< 0.00001	0.34	-	-	-
	Toll-like receptor signaling pathway	< 0.00001	0.29	-	-	-
	VEGF signaling pathway	< 0.00001	0.24	-	-	-
	Notch signaling pathway	< 0.00001	0.24	-	-	-
	Porphyrin and chlorophyll metabolism	< 0.00001	0.23	-	-	-
	PPAR signaling pathway	< 0.00001	0.18	-	-	-
	Cysteine and methionine metabolism	< 0.00001	0.16	-	-	-
	Long-term depression	< 0.00001	0.14	-	-	-
**Fema**	N-Glycan biosynthesis	-	-	< 0.00001	0.55	-
	Spliceosome	-	-	< 0.00001	0.54	-

Amino sugar and nucleotide sugar metabolism	-	-	< 0.00001	0.4	-
Hypertrophic cardiomyopathy (HCM)	-	-	< 0.00001	0.31	-
Taste transduction	-	-	< 0.00001	0.31	-
Glyoxylate and dicarboxylate metabolism	-	-	< 0.00001	0.27	-
Regulation of autophagy	-	-	< 0.00001	0.25	-
Proteasome	-	-	< 0.00001	0.24	-
Arachidonic acid metabolism	-	-	< 0.00001	0.23	-
Fc epsilon RI signaling pathway	-	-	< 0.00001	0.22	-

As the sex-shared pathways, *MAPK signaling pathway* and *p53 signaling pathway* are reported as significant in GBM. The hyperactivation of the *MAPK signaling pathway* plays a key role in GBM, and the progression of the glioma can be restricted by the inhibition of the *MAPK signaling pathway* [1]. The *p53 signaling pathway* suppresses the activity of enzyme ubiquitin specific peptidase 7 (USP7) in glioma [26]. In a male-enriched pathway, *Notch signaling pathway* in hypocretin-1-treated cells in GBM is crucially downregulated such that the inhibition of the *Notch signaling pathway* allows the hypocretin-1 to exert the antitumor effect on GBM [8]. As a pathway enriched in females, the dysregulation of key proteins associated with the *Spliceosome* may affect the Temozolomide (TMZ) resistance, a reason why GBM treatment fails, which are related to prognosis of GBM patients [25].

Supplementary Table 6. The top-ranked genes of GSE172367

	Gene	Chr	Male		Female		Score (×1e-6)	Interaction *p*-value
			p	Score (×1e-6)	*p*	Score (×1e-6)		
**Sex-shared**	PIK3R1	5	< 0.00001	1.07	< 0.00001	1.99	1.99	0.176
	HLA-G	6	< 0.00001	1.79	< 0.00001	0.98	1.79	0.181
	HLA-DMA	6	< 0.00001	2.08	< 0.00001	1.74	1.74	0.337
	HLA-C	6	< 0.00001	1.56	< 0.00001	1.17	1.56	0.109
	AKT3	1	< 0.00001	1.53	< 0.00001	0.64	1.53	0.339
	IKBKB	8	< 0.00001	0.35	< 0.00001	1.49	1.49	0.930
	CALML5	10	< 0.00001	0.61	< 0.00001	1.46	1.46	0.453
	GSK3B	3	< 0.00001	1.34	< 0.00001	0.84	1.34	0.855
	PRKACB	1	< 0.00001	1.18	< 0.00001	0.80	1.18	0.371
	AKT2	19	< 0.00001	1.09	< 0.00001	1.14	1.14	0.339
**Male-specific**	PIK3CD	1	< 0.00001	2.79	-	-	-	0.433
	MAPK3	16	< 0.00001	1.69	-	-	-	0.339
	TGFB1	19	< 0.00001	1.52	-	-	-	0.317
	MAPK1	22	< 0.00001	1.33	-	-	-	0.490
	IL1B	2	< 0.00001	1.06	-	-	-	0.795
	HLA-A	6	< 0.00001	1.06	-	-	-	0.524
	RAF1	3	< 0.00001	1.04	-	-	-	0.855
	TGFB2	1	< 0.00001	1.01	-	-	-	0.634
	YWHAQ	2	< 0.00001	0.93	-	-	-	0.766
	MAPK14	6	< 0.00001	0.92	-	-	-	0.863
**Female-specific**	PIK3CB	3	-	-	< 0.00001	3.48	-	0.733
	MAP2K2	19	-	-	< 0.00001	3.16	-	0.866
	PIK3R3	1	-	-	< 0.00001	3.01	-	0.610
	ALDH3A1	17	-	-	< 0.00001	2.75	-	0.425
	MAPK8	10	-	-	< 0.00001	2.72	-	0.980
	ITGB4	17	-	-	< 0.00001	2.39	-	0.886
	ALDH7A1	5	-	-	< 0.00001	2.35	-	0.190
	PRKAG1	12	-	-	< 0.00001	2.24	-	0.445
	CCL5	17	-	-	< 0.00001	2.24	-	0.454

GLUD1	10	-	-	< 0.00001	2.05	-	0.410

For the sex-shared genes, *PIK3R1* is related to the asthma severity with its higher expression in peripheral blood mononuclear cells [15]. *HLA-G* is an immunomodulatory factor in asthma [12]. In the 3‘UTR segment of the *HLA-G* gene, the variation sites, +3010C/G and +3142C/G, are separately concerned in asthma severity [2]. *IKBKB*’s SNPs in children increase susceptibility to the development of wheezing that may result in possibly subsequent asthma [5]. As the male-specific genes, *TGFB1* is reported in asthma airway inflammation, remodeling and cytokine [4], and decline in lung function [10]. *MAPK1* suppresses the Th2 inflammation in airway epithelial cells of allergic asthma [21]. *IL1B* upregulation appears in neutrophilic asthma [17], and high expressions of *IL1B* related to neutrophilic inflammation are involved in inferior lung function and raised chronic obstructive pulmonary disease (COPD) severity [3]. For the female-specific genes, *ALDH3A1* is identified as a potential marker to predict the diagnosis of difficult-to-control asthma [18]. *ITGB4*’s deficiency activates airway inflammation, which is a significant incentive for bipolar disorder (BD)-like behavior during asthma pathogenesis [7].

Supplementary Table 7. The top-ranked pathways of GSE172367

	Pathway	Male		Female		Score (×1e-4)
		p	**Score** (×1e-4)	***p***	**Score** (×1e-4)	
**Sex-shared**	Insulin signaling pathway	< 0.00001	4.94	< 0.00001	5.53	5.53
	Cell cycle	< 0.00001	3.91	< 0.00001	4.20	4.20
	Progesterone-mediated oocyte maturation	< 0.00001	2.99	< 0.00001	3.44	3.44
	Jak-STAT signaling pathway	< 0.00001	2.88	< 0.00001	3.40	3.40
	Arginine and proline metabolism	< 0.00001	2.66	< 0.00001	2.99	2.99
	Hypertrophic cardiomyopathy (HCM)	< 0.00001	2.61	< 0.00001	2.93	2.93
	Graft-versus-host disease	< 0.00001	2.68	< 0.00001	2.86	2.86
	Alzheimer's disease	< 0.00001	2.54	< 0.00001	2.82	2.82
	Fc gamma R-mediated phagocytosis	< 0.00001	2.52	< 0.00001	2.79	2.79
	Vascular smooth muscle contraction	< 0.00001	2.32	< 0.00001	2.74	2.74
**Male-specific**	Apoptosis	< 0.00001	0.53	-	-	-
	Cardiac muscle contraction	< 0.00001	0.47	-	-	-
	Allograft rejection	< 0.00001	0.36	-	-	-
	Base excision repair	< 0.00001	0.07	-	-	-
	Maturity onset diabetes of the young	< 0.00001	0.06	-	-	-
**Female-specific**	Focal adhesion	-	-	< 0.00001	1.64	-
	Ubiquitin mediated proteolysis	-	-	< 0.00001	0.97	-
	B cell receptor signaling pathway	-	-	< 0.00001	0.73	-
	Tight junction	-	-	< 0.00001	0.38	-

Taste transduction	-	-	< 0.00001	0.33	-
Cysteine and methionine metabolism	-	-	< 0.00001	0.24	-

In the sex-shared pathways, regarding the regulation of the *JAK-STAT signaling pathway*, pyrroloquinoline quinone (PQQ) mitigates allergic airway inflammation in mice. PQQ is a potential therapeutic agent for inflammatory diseases, including asthma [14]. *JAK-STAT signaling pathway* allows the exposure of perfluorooctanesulfonate (PFOS) and perfluorooctanoate (PFOA) to induce airway inflammation of asthma. *JAK-STAT6 signaling pathway*, a key member of *JAK-STAT*, engages in several asthma stages [24]. The enzyme arginase of the *Arginine and proline metabolism pathway*, relevant to asthma pathogenesis, increases activity in the serum of asthmatic individuals [16]. For the pathway enriched in males, Panax Notoginseng Saponins R1 (PNS-R1) alleviates Dexamethasone (Dex)-induced apoptosis in bronchial epithelial cells by the inhibition of mitochondrial *Apoptosis* pathway, highlighting the potential of the PNS-R1 gene in asthma treatment [28]. As the female-enriched pathway, *Ubiquitin mediated proteolysis* pathway might contribute to the development of asthma and the credible therapeutic approaches of asthma diagnosis and treatment in the future [9]. Upregulated expression in hypomethylation of genes in *Ubiquitin mediated proteolysis* may describe increased abundance or phenotypic differences of monocytes in asthma [6].

Supplementary Table 8. The details of TCGA datasets

Data	Tissue Type	# Gene	# Sample	# Male	# Female
LIHC	liver hepatocellular carcinoma	4,360	372	251	121
STAD	stomach adenocarcinoma	4,369	407	261	146
LUAD	lung adenocarcinoma	4,365	508	237	271
LUSC	lung squamous cell carcinoma	4,366	495	366	129
GBM/LGG	glioblastoma multiforme/brain lower grade glioma	4,350	693	397	296

Supplementary Table 9. The details of Asthma datasets

Data	Tissue Type	# Gene	# Sample	# Male	# Female
GSE8052	lymphoblastoid cell line	4,394	404	221 (C - 58, A - 163)	183 (C - 78, A - 105)
GSE172367	airway epithelial cell	3,019	190	112 (C - 64, A - 48)	78 (C - 42, A - 36)

References

1. Sevin ¸c Ak ¸cay, Emine G ̈uven, Muhammad Afzal, and Imran Kazmi. Non-negative matrix factorization and differential expression analyses identify hub genes linked to progression and prognosis of glioblastoma multiforme. Gene, 824:146395, 2022.
2. Cinthia C Alves, Luísa KP Arruda, Fabíola R Oliveira, Juliana D Massaro, Beatriz J Aquino, Michelle A Paz, Erick C Castelli, Celso T Mendes-Junior, and Eduardo A Donadi. Human leukocyte antigen-g 3’untranslated region polymorphisms are associated with asthma severity. Molecular immunology, 101:500–506, 2018.
3. Katherine J Baines, Netsanet A Negewo, Peter G Gibson, Juan-Juan Fu, Jodie L Simpson, Peter AB Wark, Michael Fricker, and Vanessa M McDonald. A sputum 6 gene expression signature predicts inflammatory phenotypes and future exacerbations of copd. International Journal of Chronic Obstructive Pulmonary Disease, pages 1577–1590, 2020.
4. Sandra Dragicevic, Katarina Milosevic, Branimir Nestorovic, and Aleksandra Nikolic. Influence of the polymorphism c-509t in the tgfb1 gene promoter on the response to montelukast. Pediatric Allergy, Immunology, and Pulmonology, 30(4):239–245, 2017.
5. Susanna Esposito, Valentina Ierardi, Cristina Daleno, Alessia Scala, Leonardo Terranova, Claudia Tagliabue, Walter Peves Rios, Claudio Pelucchi, and Nicola Principi. Genetic polymorphisms and risk of recurrent wheezing in pediatric age. BMC Pulmonary Medicine, 14(1):1–10, 2014.
6. Lakshitha P Gunawardhana, Peter G Gibson, Jodie L Simpson, Miles C Benton, Rodney A Lea, and Katherine J Baines. Characteristic dna methylation profiles in peripheral blood monocytes are associated with inflammatory phenotypes of asthma. Epigenetics, 9(9):1302–1316, 2014.
7. Li Han, Leyuan Wang, Sha Tang, Lin Yuan, Shuangyan Wu, Xizi Du, Yang Xiang, Xiangping Qu, Huijun Liu, Huaiqing Luo, et al. Itgb4 deficiency in bronchial epithelial cells directs airway inflammation and bipolar disorder-related behavior. Journal of Neuroinflammation, 15(1):1–14, 2018.
8. Renzheng Huan, Jianhe Yue, Jinhai Lan, Jia Wang, Yuan Cheng, Jiqin Zhang, and Ying Tan. Hypocretin-1 suppresses malignant progression of glioblastoma cells through notch1 signaling pathway. Brain Research Bulletin, 196:46–58, 2023.
9. Zhi-Jian Huang, Qin-Hai Shen, Yan-Sheng Wu, and Ya-Li Huang. A gibbs sampling method to determine biomarkers for asthma. Computational Biology and Chemistry, 67:255–259, 2017.
10. Gyu Young Hur and David H Broide. Genes and pathways regulating decline in lung function and airway remodeling in asthma. Allergy, asthma & immunology research, 11(5):604–621, 2019.
11. Hang Ji, Yixu Ba, Shuai Ma, Kuiyuan Hou, Shan Mi, Xin Gao, Jiaqi Jin, Qin Gong, Ting Liu, Fang Wang, et al. Construction of interferon-gamma-related gene signature to characterize the immune-

inflamed phenotype of glioblastoma and predict prognosis, efficacy of immunotherapy and radiotherapy. Frontiers in immunology, 12:729359, 2021.

12. Jinyan Li, Yuqiu Hao, Wei Li, Xuejiao Lv, and Peng Gao. Hla-g in asthma and its potential as an effective therapeutic agent. Allergologia et Immunopathologia, 51(1):22–29, 2023.
13. Tianwen Li, Zhipeng Yang, Haoyuan Li, Jingjing Zhu, Ye Wang, Qisheng Tang, and Zhifeng Shi. Phospholipase cγ1 (plcg1) overexpression is associated with tumor growth and poor survival in idh wild-type lower-grade gliomas in adult patients. Laboratory Investigation, 102(2):143–153, 2022.
14. Scott M Lundberg and Su-In Lee. A unified approach to interpreting model predictions. Advances in neural information processing systems, 30, 2017.
15. Zhihui Min, Jiebai Zhou, Ruolin Mao, Bo Cui, Yunfeng Cheng, Zhihong Chen, et al. Pyrroloquinoline quinone administration alleviates allergic airway inflammation in mice by regulating the jak-stat signaling pathway. Mediators of Inflammation, 2022, 2022.
16. Yan Qian, Yun Sun, Yi Chen, Zhengdao Mao, Yujia Shi, Di Wu, Bin Gu, Zhiguang Liu, and Qian Zhang. Nrf2 regulates downstream genes by targeting mir-29b in severe asthma and the role of grape seed proanthocyanidin extract in a murine model of steroid-insensitive asthma. Pharmaceutical Biology, 60(1):347–358, 2022.
17. Kevin D Quinn, Michaela Schedel, Yasmeen Nkrumah-Elie, Anthony Joetham, Michael Armstrong, Charmion Cruickshank-Quinn, Richard Reisdorph, Erwin W Gelfand, and Nichole Reisdorph. Dysregulation of metabolic pathways in a mouse model of allergic asthma. Allergy, 72(9):1327–1337, 2017.
18. Stephany S´anchez-Ovando, Katherine J Baines, Daniel Barker, Peter A Wark, and Jodie L Simpson. Six gene and th2 signature expression in endobronchial biopsies of participants with asthma. Immunity, inflammation and disease, 8(1):40–49, 2020.
19. Wenping Song, Si Zheng, Meng Li, Xia Zhang, Rui Cao, Cheng Ye, Rongguang Shao, Guangxi Li, Jiao Li, Shigang Liu, et al. Linking endotypes to omics profiles in difficult-to-control asthma using the diagnostic chinese medicine syndrome differentiation algorithm. Journal of Asthma, 57(5):532–542, 2020.
20. Aglaia Vignoli, Elena Lesma, Rosa Maria Alfano, Angela Peron, Giulia Federica Scornavacca, Maura Massimino, Elisabetta Schiavello, Silvia Ancona, Michele Cerati, Short Article Title 3 Gaetano Bulfamante, et al. Glioblastoma multiforme in a child with tuberous sclerosis complex. American Journal of Medical Genetics Part A, 167(10):2388–2393, 2015.
21. Yulin Wang, Weijiang Zhao, Zhe Xiao, Gefei Guan, Xin Liu, and Minghua Zhuang. A risk signature with four autophagy-related genes for predicting survival of glioblastoma multiforme. Journal of Cellular and Molecular Medicine, 24(7):3807–3821, 2020.

22. T Xia, J Ma, Y Sun, and Y Sun. Androgen receptor suppresses inflammatory response of airway epithelial cells in allergic asthma through mapk1 and mapk14. Human & Experimental Toxicology, 41:09603271221121320, 2022.

23. Xin Xia, Xixi Li, Fanying Li, Xujia Wu, Maolei Zhang, Huangkai Zhou, Nunu Huang, Xuesong Yang, Feizhe Xiao, Dawei Liu, et al. A novel tumor suppressor protein encoded by circular akt3 rna inhibits glioblastoma tumorigenicity by competing with active phosphoinositide-dependent kinase-1. Molecular cancer, 18:1–16, 2019.

24. Dan-Dan Xiong, Wen-Qing Xu, Rong-Quan He, Yi-Wu Dang, Gang Chen, and Dian-Zhong Luo. In silico analysis identified mirna-based therapeutic agents against glioblastoma multiforme. Oncology reports, 41(4):2194–2208, 2019.

25. Mo Yang, Li-Yue Li, Xiao-Di Qin, Xiao-Yan Ye, Shu Yu, Qing Bao, Lin Sun, Zhi-Bin Wang, Michael S Bloom, Pasi Jalava, et al. Perfluorooctanesulfonate and perfluorooctanoate exacerbate airway inflammation in asthmatic mice and in vitro. Science of The Total Environment, 766:142365, 2021.

26. Guo-zhong Yi, Wei Xiang, Wen-yan Feng, Zi-yang Chen, Yao-min Li, Sheng-ze Deng, Man-lan Guo, Liang Zhao, Xue-gang Sun, Min-yi He, et al. Identification of key candidate proteins and pathways associated with temozolomide resistance in glioblastoma based on subcellular proteomics and bioinformatical analysis. BioMed Research International, 2018, 2018.

27. Lei Yi, Yan Cui, Qingfu Xu, and Yugang Jiang. Stabilization of lsd1 by deubiquitinating enzyme usp7 promotes glioblastoma cell tumorigenesis and metastasis through suppression of the p53 signaling pathway. Oncology reports, 36(5):2935–2945, 2016.

28. Shuchang Zhou, Rui Niu, Han Sun, Sung-Hak Kim, Xiong Jin, and Jinlong Yin. The map3k1/c-jun signaling axis regulates glioblastoma stem cell invasion and tumor progression. Biochemical and Biophysical Research Communications, 612:188–195, 2022.

29. Wenjing Zou, Chao Niu, Zhou Fu, and Caihui Gong. Pns-r1 inhibits dex-induced bronchial epithelial cells apoptosis in asthma through mitochondrial apoptotic pathway. Cell & Bioscience, 9(1):1–10, 2019.

30. Chen, Jianbin, et al. "Genomic landscape of lung adenocarcinoma in East Asians." Nature genetics 52.2 (2020): 177-186.
